# Assessment of Dietary Diversity of Mothers and Children of 6–24 Months from Eastern and Southern Provinces of Zambia

**DOI:** 10.1155/2019/1049820

**Published:** 2019-07-03

**Authors:** Emmanuel Oladeji Alamu, Therese Gondwe, Toluwalope Emmanuel Eyinla, Busie Maziya-Dixon

**Affiliations:** ^1^Food and Nutrition Sciences Laboratory, International Institute of Tropical Agriculture Southern Africa Research and Administration Hub (SARAH) Campus, Chelston, Lusaka, Zambia; ^2^Food and Nutrition Sciences Laboratory, International Institute of Tropical Agriculture (IITA), Ibadan, Oyo State, Nigeria

## Abstract

In-depth information on dietary diversity and food consumption patterns in Zambian households is still scarce. This study, therefore, probed dietary intakes of mothers and their children living in households of two Zambian districts, Chipata and Monze, located in the eastern and southern provinces of Zambia, respectively. After assessing their diet, Dietary Diversity Scores (DDSs) were calculated and classified into low and high categories, while correlations were used to test determinants of DDS. The assessment revealed that the consumption of cereal-based products ranked highest in frequency. Specifically, the consumption of maize-based foods was highest in Chipata (55.43%) and then in Monze (43.56%) households. There was an observed low preference for mixed dishes that were not either maize or groundnut porridges. We also found positive and negative correlations of DDS with the educational level of household heads and age of mothers, respectively. We, therefore, suggest that increased nutrition education may improve dietary preferences, so also further investigation into other factors hindering low choices for mixed recipes will be useful in increasing overall diet quality.

## 1. Introduction

Food consumption data are essential sources for tracking information on household food insecurity and nutrition outcomes, especially when the quality of diet is highlighted. One of the key elements of assessing diet quality of populations is to measure the variety and type of their consumed foods [1]. This variety of food consumption is usually referred to as dietary diversity. Dietary diversity is a qualitative measure of food consumption that shows nutrient adequacy of the diet of individuals and households [2, 3]. Considering that over 40 nutrients are needed in the human diet for best nutrition and well-being [4], a different combination of foods from various food groups is required to help meet individual nutritional requirements and promote good health [2, 5]. Measuring dietary diversity has been found as a useful tool for the rapid assessment of food security and nutritional status in low-income settings [6, 7]. Increasing dietary diversity is a proven intervention that improves nutrient adequacy in children aged 6 months to 2 years [2]. Nutrient-rich foods from different diets are essential elements in child feeding that support dietary needs and adequate growth during their early years of life. Dietary diversity has been established as a significant predictor of growth, as illustrated by an analysis of Demographic and Health Survey data from children aged 6–24 months in 11 countries in Africa and Latin America [3]. Intake of a diverse variety of foods has been a recommendation for achieving adequate nutrient intake, and the advice appears in the dietary guidelines of many countries. Several factors can hinder diversification of diets, especially among poor populations of developing countries.

In most cases, it hinges on the availability of food and the corresponding economic or physical access. While there is evidence that socioeconomic constraints can affect eating a varied diet [8, 9], it is unclear if the sociodemographic characteristics of a population influence the choice of food and dietary diversity. A survey evaluating the nutrition and food diversity among smallholder farmers found high food insecurity and low crop and diet diversity among farmers three months after harvest [10]. There is, however, an association between improved child dietary diversity and increased maternal nutritional diversity knowledge and practice [10]. The studies were either limited in sampling only some regions or targeted farming households only and did not probe the diversification of diets in mother-child pairs. This study, therefore, aimed to assess mother-child pairs with a focus on dietary intake, food frequency, and associated factors affecting dietary in children aged 6–24 months in Chipata and Monze districts in the Eastern and Southern provinces of Zambia, respectively.

## 2. Methodology

### 2.1. Study Design

A cross-sectional study design was used, and households with children aged 6–24 months were randomly selected from the database of children (<5 yrs) registered at the respective district clinic; a total of 200 households were selected from each of the two districts making a total of 400 households. This selection was based on random sampling with the criteria being voluntary participation. A structured questionnaire was used to collect information on household demographics and food consumption twice at 3-month intervals.

### 2.2. Sampling and Sample Size

The sampling unit for the study was households with children aged 6 to 24 months. The youngest child within the household was selected, and if there were more than a mother/caregiver in the household, the most senior was selected. The two districts were chosen purposely, due to their high stunting levels, and the eight camps from each district were randomly selected. The selected households (400) for the study were verified and registered at 16 camps from both Chipata and Monze districts in Eastern and Southern Zambia, respectively. The households were accorded household numbers purposely for first and subsequent data collections. The eight camps from Chipata were coded 01 to 08 and those from Monze camps were coded 09 to 16. Each household was assigned a unique household number.

### 2.3. Data Collection

The trained local field workers performed data collection with a pretested questionnaire. A total of 16 field workers from the two districts were trained on the 24-hour multipass recall dietary assessment tool. Data on dietary intake were assessed using the multipass 24-hour dietary recall method. The interview was repeated twice at 3-month intervals. Field workers carried various samples of foods, scales, spoons, cups, small food containers, etc. and used these to estimate amounts consumed. The data collected for the two periods were used to generate dietary diversity scores (DDSs) as a measure of diet quality. The mothers supplied the dietary intake information and pattern for the children. Total DDSs amounted to a minimum of one to a maximum of eight. From the repeated dietary recall, the number of times a food was consumed was taken as a single count. The different foods were accounted for, and the portion sizes averaged.

### 2.4. Ethics

Ethical clearance approval was obtained from the University of Zambia Biomedical Research Ethics Committee (UNZABREC) with assurance no. FWA00000338 and approval no. IRB00001131 of IORG0000774, and the final authority was obtained from the Ministry of Health. The mother of the selected child was informed about the nature of the study. Respondent participation in the study was voluntary with voluntary informed consent requested from households. Questionnaires were administered after the families agreed to participate in the study.

## 3. Data Processing and Statistical Analysis

The data were analysed using SAS version 9.3. Food frequency, amounts of foods consumed (g), and DDS were calculated by summing the number of unique food groups consumed by the child in the 24-hour period. Pearson's correlations analysis of DDS and sociodemographic characteristics were run. A food frequency of 10 was used as the cutoff point, which accounted for 95% of the food consumed by the participants. Mean ± SD value for the weight of each food variety consumed was computed. The classification of the Dietary Diversity Score (DDS) into low and high categories was adapted from [2] with slight modifications. The ranges used were 1 to 4 foods for Low DDS and 5 to 8 foods for High DDS.

## 4. Results

### 4.1. Sociodemographic Characteristics of Respondents: Households' Heads and Mothers

The mean age of household heads selected for this study was 35.6 ± 11.72 years (range 17–86 years) for Chipata and 40.4 ± 15.8 years (range 17–94 years) for Monze, while the mean age for mothers was 26.9 ± 8.17 years (range 15–50 years) for Chipata and 27.20 ± 7.95 years (range 15–49 years) for Monze (Table 1). Table 1 also shows the marital status and educational levels of household heads (HHs) and mothers. About 40.57% (Chipata, *n*=284) and 28.57% (Monze, *n*=200) of HH were married monogamously, while 6.86% (Chipata, *n*=48) and 8.71% (Monze, *n*=61) married polygamously. Mothers that participated in this study were married monogamously (Chipata, 38%, *n*=266; Monze, 25%, *n*=175) or married polygamously (Chipata, 3.86%, *n*=27; Monze, 7.43%, *n*=52).

The educational status of the respondents shows that most of the HHs had not completed primary school (Chipata, 19.43%, *n*=136; Monze, 8.71%, *n*=61) or had completed primary school (Chipata, 15.14%, *n*=106; Monze, 12.43%, *n*=87). Some HHs from Monze (16.71%) had not completed secondary education. Only 0.14% and 0.29% had completed university education in Chipata and Monze districts, respectively. However, the educational status of mothers was like that of HHs. Most mothers had not completed primary school (Chipata, 23%, *n*=161; Monze, 10.86%, *n*=76), some had completed primary school (Chipata, 12.71%, *n*=89; Monze, 11.86%, *n*=83), and others had not completed secondary school (Chipata, 11.43%, *n*=80; Monze, 18.86%, *n*=132). About 8.43% and 1.29% had no education at all in Chipata and Monze districts, respectively. Most of the HHs and mothers from the households used for this study had a low level of education, and this could have a major impact on the selection of foods consumed by households.

### 4.2. Household Consumption of Common Foods (Maize-Based and Groundnut-Based) in Chipata and Monze

Table 2 presents information on the household consumption of maize-based foods in Chipata and Monze. About 55.43% (*n*=383) and 43.56% (*n*=301) of households consumed maize-based foods daily across the two districts. Most households consumed mostly maize + groundnut porridge (Chipata, 53.66%, *n*=389; Monze, 39.31%, *n*=285) and plain maize porridge (Chipata, 44.97%, *n*=326; Monze, 37.52%, *n*=272). However, the least consumed of all maize-based foods were maize + bean porridge (Chipata, 1.66%, *n*=12; Monze, 4.69%, *n*=34) and maize + fish porridge (Chipata, 0.41%, *n*=3; Monze, 2.76%, *n*=20). This study shows that most households did not consume maize-based foods fortified with other sources of protein (beans and fish). Moreover, groundnut-based recipes were consumed daily by Chipata respondents (22.21%, *n*=157) compared to 6.65% (*n*=47) recorded for Monze respondents, while 20.51% (*n*=145) and 22.49% (*n*=159) consumed it only 2 to 3 times daily for Chipata and Monze, respectively (Table 2). A preference for groundnut + leafy vegetables was observed to have the highest positive response of 48.69% and 39.17% in Chipata and Monze districts, respectively. The consumption of porridge made from combinations of groundnut with staple crops, such as rice, cassava, and sweet potato, was very low. The major foods consumed were maize + groundnut based, and this could be because they are the major crops grown in the two districts and thus formed a major part of their diet.

### 4.3. Food Frequency (*N*) and Weight (Grams) of Foods Consumed by Mothers and Children

The food frequency and weight of foods consumed by children from Chipata and Monze districts are presented in Tables 3 and 4. From the dietary intake assessment, seven categories of food were found: cereals and cereal-based products, legumes and legume-based products, starchy roots and tubers, fruits and vegetables, meat and fish products, eggs and egg products, and milk and milk products. The most commonly consumed food category was the cereals and cereal-based products followed by fruits and vegetables, even though the latter category had a greater variety of foods. Nshima and porridge (maize-based foods) were the most frequently consumed foods by children from the two districts with consumption of 130.1 ± 82.86 g/day (*n*=214) and 150.7 ± 81.56 g/day (*n*=209) for Chipata and 121.9 ± 65.20 g/day (*n*=159) and 154.3 ± 82.40 (*n*=145) for Monze, respectively. Green leafy vegetables such as pumpkin leaves (88.5 ± 89.36 g/per day, *n*=137), rape relish (45.9 ± 49.23 g/day, *n*=84), and rape (82 ± 72.23 g/day, *n*=35) were the most commonly consumed among children from Chipata while children from Monze District consumed cheele (216.4 ± 116.36 g/day, *n*=57) and rape (48.2 ± 28.18 g/day, *N*=51) with a median intake of 204 g and 40 g per day, respectively. The consumption of major sources of animal protein (meat and fish products, eggs and egg products, and milk and milk products) was observed to be very low for Chipata (*n*=12 to 21) and Monze (*n*=12 to 13). A small number of children from Chipata was fed with breast milk (*n*=18) the day preceding the dietary intake assessment and none from Monze, but sour milk (*n*=12) was found to be fed to children from both districts.

The food frequency and weight (grams) of food consumed by mothers from Chipata and Monze districts are summarised in Tables 5 and 6. The distribution and category of foods were like those obtained for the children, with the fruit and vegetable group having the highest number of food varieties (10 to 13 types) compared to other food groups. Mothers from Chipata had more foods under the legume and legume products and fruit and vegetable food groups compared to their Monze counterparts. Nshima and porridge were the most consumed cereal-based product with a mean portion size of 602.1 ± 248.09 g/day (*n*=218) and 371.9 ± 182.78 g/day (*n*=142) for Chipata mothers, while nshima (650.2 ± 288.28 g/day, *n*=177) and samp (515.5 ± 235.54 g/day, *n*=119) were mostly consumed by mothers in Monze. This study shows that nshima (maize dough) was the major food for mothers in Chipata, but samp (maize grits) was found to be the major food consumed among Monze mothers.

The fruit and vegetable food group was the second most consumed by mothers, with pumpkin leaves (198 ± 109.84 g/day, *n*=172), rape (151.2 ± 65.72 g/day, *n*=144), and okra (145.1 ± 69.53 g/day, *n*=59) being their favourite vegetables in Chipata, while rape (128.1 ± 56.03 g/day, *n*=68) and okra (145.1 ± 69.53 g/day, *n*=59) were the favourite vegetables for mothers from Monze. This was similar to fish products. Although meat and fish products consumption was deficient, those consumed included kapenta, fish, chicken, fish relish, and pork.

Food drinks were found to be a food group for mothers, not for children. The daily intake of these drinks (sweet beer and chibwantu) was very high, although the frequency was low. Sweet beer (519 ± 248.75 g/day, *n*=61) and chibwantu (980.2 ± 333 g/day, *n*=10) for Chipata mothers, and for Monze mothers, sweet beer (568.5 ± 330.2 g/100 g, *n*=82) and chibwantu drink (671.6 ± 405.68 g/100 g, *N*=14) were observed.

### 4.4. Dietary Diversity Scores (DDSs) for Mothers and Children

The DDS of children across the two districts is presented in Table 7. The mean DDS for the children was 4.1 ± 1.38 (range 1 to 8), but males had 4.1 ± 1.42 and females had 4.1 ± 1.32 with no significant difference seen (*P* < 0.05). Most (62.69%, *n*=247) of the children consumed food items from 1 to 4 food groups, and only 37.31% (*n*=147) consumed a diversified diet from 5 to 8 food groups showing low dietary diversity (LDD) and high dietary diversity (HDD), respectively. However, 27.92% (*n*=110) consumed 4 food groups showing mid-dietary diversity (MDD), while 0.76% (*n*=3) with HDD consumed most of the 8 food groups. The children from Monze district had a slightly higher DDS (4.4 ± 1.34) than that of children in Chipata district (3.9 ± 1.37). However, 26.27% and 27.19% of children from Chipata had a DDS of 3 and 4, respectively, while children from Chipata had a DDS of 4 (8.81%) and 5 (25.99%). Comparatively, children from Chipata had a higher number of respondents (70.05%) in the low category (DDS 1 to 4) than their Monze counterparts (53.67%). Many more children from Monze district (46.33%) were in the high DDS (5 to 8) category compared to those in Chipata district (29.95%).

Table 7 also shows the distribution of DDS of mothers from Chipata and Monze districts. The mean DDS of mothers (4.8 ± 1.33, *n*=396) was slightly above the low cutoff (4.0), thus indicating that most women consumed foods from >4 different groups. However, mothers from Chipata had a mean DDS (range 2 to 8) of 5.1 ± 1.47 (*n*=219) with the majority (64.38%) in the high category of DDS (5 to 8), while those from Monze had 4.6 ± 1.08 (*n*=177) with 50.85% in the low class of DDS (1 to 4). The frequency of the scores shows that no mother had a DDS of 1 across the districts, but the highest number of mothers (26.94%) from Chipata was found to have a DDS of 5 and Monze (36.16%) had a DDS of 4. No woman from Monze District had a DDS of 8, but 5% (*n*=11) was recorded for Chipata, and this shows that mothers from Chipata consumed a more diversified diet than their Monze counterparts. The observation was contrary to what was observed among the children, where a higher percentage of Monze children consumed a diet from more food groups than those from Chipata.

### 4.5. Correlation of DDS and Sociodemographic Characteristics of Household Heads (HHs) and Mothers

The correlation coefficient (*r*) of DDS (Chipata and Monze) and sociodemographic characteristics of HHs and mothers are presented in Table 8. There was a significant positive correlation between the DDS and educational levels of HHs in Chipata (*r* = 0.15268, *P* < 0.01) and Monze (*r* = 0.15271, *P* < 0.05). The DDS of mothers sampled from Chipata was correlated and significant with their educational level (*r* = 0.21265, *P* < 0.001) and age (*r* = −0.16728, *P* < 0.01). However, the age of HHs was correlated and significant (*r* = 0.15209, *P* < 0.05) with DDS among Monze respondents, and this shows that the level of education of the HHs, education level, and age of mothers influenced the diet diversity of food consumed by the households. Of note is the negative correlation between DDS and age of the mothers sampled from Chipata (*r* = –0.16728, *P* < 0.01) and Monze (*r* = −0.08110, *P* < 0.01), and this could mean that improved knowledge through education in younger women compared to older women in the sampled districts contributed to the choice of foods consumed.

## 5. Discussion

Dietary intake assessment was carried out using a quantitative 24-hour dietary recall to generate a frequency of consumption, portion sizes, and Dietary Diversity Scores (DDSs) of foods commonly consumed by mothers and children in the sampled districts. The mean Dietary Diversity Score (DDS) calculated in this study shows a low diversity of foods for both mothers and their children, which infers that majority of households consume a monotonous diet that focuses on a limited number of food groups. This DDS suggests a low diet quality as described by Nupo et al. [5] and Vandevijvere et al. [11]. The numbers of foods available for children and mothers in Chipata were more than that of foods available for their Monze counterparts, and this was evident in the DDS of the mothers from Chipata who had a higher DDS. It was not the same as children from Chipata who had a low DDS. This may suggest that availability may not necessarily indicate consumption of foods by the children. The scores of the children from both districts fell below the WHO infant and young child feeding indicator on dietary diversity, which suggests greater or equal to 4 food groups/day [12]. Thus, nutrition education on the benefits of diversifying diets may be useful to improve the nutrient intake of the mothers and children from Chipata and Monze. The high consumption of cereal-based foods and assorted vegetables are in concordance with similar studies and surveys carried out among Zambian populations [13, 14]. Furthermore, as discussed by Doko et al. [15] and Mamiro et al. [16], cereals and their products are the main staples of populations in Eastern and Southern Africa. This is particularly so in the mother-child pairs in this study, where maize-based products (nshima and maize porridge) were the most frequently consumed foods. Frequency of consumption of animal source foods was very low and was restricted to milk, fish, and chicken and their products. There are more diverse sources of dietary energy intake for children in Chipata which were obtained mainly from cereals and starchy roots and tubers, but there were fewer sources of dietary energy among the Monze children. A similar level of diversity is observed in the foods that chiefly supply protein to children from Chipata, relying on three sources compared to only beans consumed by children from Monze. This dependence on plant sources of foods can have implications on micronutrient intake, which usually results in severe undernutrition [17]. There is evidence [18], which proves that dietary diversity, as an indicator of micronutrient adequacy, is associated with nutritional outcomes of infants in a Zambian population. These deficiencies were also established in mother-child pairs assessed in the northern provinces of Zambia [13]. An improvement in dietary diversification presents short- and long-term benefits. Even though children in Chipata had more types of foods within groups, their DDS was lower than their counterparts from Monze District, and this could be because of poor knowledge of value addition to staple crops. As observed from the study, responses on whether some recipes were consumed turned out to be negative. In the absence of nutrient-dense foods of animal origin, an understanding of how to maximize available crops for nutrition security could help in improving the quality of the diet, especially for children with vital nutritional needs. Nutrition education, in this regard, has been established to be a high impact intervention to improve nutrient adequacy [19, 20]. According to results presented in Tables 3 and 6, which show nshima and maize porridge (both maize-based) as the foods with highest food frequency counts, and Table 2, which shows a monotonous preference for maize and groundnut porridge, it is obvious that the sampled populations are highly dependent upon maize-based foods. These observations are similar to food consumption data of Zambians, as reported by Nyirenda et al. [21]. Worthy of note is the low preferences for maize with fish porridge and maize with beans porridge. These foods can improve diversity and adequate nutrients if accepted. A feeding trial of biofortified foods in Zambian children carried out by Schmaelzle et al. [22] emphasised education on the usefulness and potential of new recipes as a missing link for poor food choices for diversity. Demographic characteristics, as highlighted, maybe for the cause of the low dietary diversity seen in the sampled populations. The results of the study, which show an association between the educational level of the household head and DDS in both districts, is a determining factor in improving the overall quality of diet in households in the sampled districts. Associations between mothers' knowledge of diet diversification with sociodemographic factors such as husband's education and age of the mother have been reported in the literature [10]. This association is a pointer to the positive impact of an individual's educational status on healthy food choices. This indicates that enlightenment about food may be needed at the household level in Zambia to encourage improved dietary practices.

## 6. Conclusion

The study has shown low diversity in the diet of mothers and children aged 6–24 months in the sampled Zambian eastern and southern provinces. While maize-based foods are often consumed, variety in the recipes is low, and the low preference for mixed foods other than maize and groundnut porridge confirms this observation. There are suggestions that nutrition education could improve this preference, and it may be beneficial to take a closer look at factors hindering value addition to maize as evident in the low choices for recipes not usually consumed. The correlation results obtained in this study are also consistent with those of other researchers who proved that insufficient higher education is a risk factor for not meeting optimum dietary diversity in young children [23]. These relationships can hurt the nutritional status of the child since, at this stage of development, food choices are limited by general household food security.

## Figures and Tables

**Table 1:** Sociodemographic characteristics of household heads (HHs) and mothers from Chipata and Monze.

Age	*N*	Mean ± SD	Min.	Max.
Age of HHs (yr)	392	35.6 ± 11.72	17	86
	304	40.4 ± 15.8	17	94
Age of mothers (yr)	392	26.9 ± 8.17	15	50
	304	27.2 ± 7.95	15	49
			Chipata percent (*N*)	Monze percent (*N*)
*Marital status*				
Head of household	Single		3.14 (22)	1 (7)
	Married, monogamously		40.57 (284)	28.57 (200)
	Married, polygamously		6.86 (48)	8.71 (61)
	Separated		0.57 (4)	1.43 (10)
	Divorced		1.43 (10)	1 (7)
	Widow(er)		3.57 (25)	3.14 (22)
Mothers	Single		10 (70)	8.43 (59)
	Married, monogamously		38 (266)	25 (175)
	Married, polygamously		3.86 (27)	7.43 (52)
	Separated		0.86 (6)	0.86 (6)
	Divorced		2.29 (16)	1.71 (12)
	Widow(er)		1.14 (8)	0.43 (3)
*Educational level*				
Head of household	None		7.29 (51)	1.86 (13)
	Primary not completed		19.43 (136)	8.71 (61)
	Primary completed		15.14 (106)	12.43 (87)
	Secondary not completed		10 (70)	16.71 (117)
	Secondary completed		3.71 (26)	3.29 (23)
	Tertiary not completed		0.14 (1)	0.14 (1)
	Tertiary completed		0.29 (2)	0.43 (3)
	University completed		0.14 (1)	0.29 (2)
Mothers	None		8.43 (59)	1.29 (9)
	Primary not completed		23 (161)	10.86 (76)
	Primary completed		12.71 (89)	11.86 (83)
	Secondary not completed		11.43 (80)	18.86 (132)
	Secondary completed		0.29 (2)	0.86 (6)
	Tertiary not completed		0.14 (1)	0.14 (1)
	University completed		0.14 (1)	0 (0)

**Table 2:** Household consumption of maize-based and groundnut based foods in Chipata and Monze districts.

	Responses	Chipata percent (*N*)	Monze percent (*N*)
*Maize-based foods*			
Frequency consumption	Daily	55.43 (383)	43.56 (301)
	2-3 times a week	0.43 (3)	0 (0)
	Rarely	0.43 (3)	0 (0)
Maize and groundnut porridge	Yes	53.66 (389)	39.31 (285)
	No	3.59 (26)	3.45 (25)
Maize and bean porridge	Yes	1.66 (12)	4.69 (34)
	No	55.59 (403)	38.07 (276)
Maize and fish porridge	Yes	0.41 (3)	2.76 (20)
	No	56.83 (412)	40 (290)
Fresh maize and fresh groundnut porridge	Yes	0.83 (6)	8.97 (65)
	No	56.41 (409)	33.79 (245)
Plain maize porridge	Yes	44.97 (326)	37.52 (272)
	No	12.28 (89)	5.24 (38)
Others	Yes	5.93 (43)	30.90 (224)
	No	51.31 (372)	11.86 (86)
*Groundnut based foods*			
Frequency of consumption	Daily	22.21 (157)	6.65 (47)
	2-3 times a week	20.51 (145)	22.49 (159)
	Weekly	2.69 (19)	3.25 (23)
	Monthly	1.41 (10)	3.68 (26)
Maize and groundnut porridge	Yes	52.55 (381)	40.14 (291)
	No	4.69 (34)	2.62 (19)
Sweet potato and groundnut porridge	Yes	6.21 (45)	6.90 (50)
	No	51.03 (370)	35.86 (260)
Rice and groundnut porridge	Yes	4.41 (32)	3.31 (24)
	No	52.83 (383)	39.45 (286)
Cassava and groundnut porridge	Yes	0 (0)	0.83 (6)
	No	57.24 (415)	41.93 (304)
Fresh maize and fresh groundnut porridge	Yes	0.83 (6)	7.72 (56)
	No	56.41 (409)	35.03 (254)
Groundnut and leafy vegetables	Yes	48.69 (353)	39.17 (284)
	No	8.55 (62)	3.59 (26)
Groundnut and nonleafy vegetables	Yes	14.62 (106)	14.9 (108)
	No	42.62 (309)	27.86 (202)
Groundnut and pumpkin porridge	Yes	10.76 (78)	2.76 (20)
	No	46.48 (337)	40 (290)
Others	Yes	1.79 (13)	18.07 (131)
	No	55.45 (402)	24.69 (179)

**Table 3:** Food frequency and weight (grams) of foods consumed by children in Chipata.

Food type	*N*	Mean ± SD	Median	Min.	Max.	CV
*Cereals and cereal-based products*						
Nshima	214	130.1 ± 82.86	117	6	764	63.7
Porridge (maize)	209	150.7 ± 81.56	132	32	552	54.1
Buns	40	44.1 ± 35.15	35	0.9	148	79.7
Rice	23	123.7 ± 77.12	109	19	348	62.3
Samp	19	92.3 ± 52.18	95	21	214	56.5
Fresh maize	10	56.2 ± 34.24	47.5	15	124	60.9
Fritter	14	41.9 ± 20.4	36.5	19	88	48.7
*Legumes and legume-based products*						
Beans	50	71.3 ± 43.34	62	16	214	60.8
Soya pieces	26	80.6 ± 67.56	68	10	330	83.8
*Starchy roots and tubers*						
Cassava	13	84.5 ± 43.57	72	20	163	51.5
Sweet potato	11	128.3 ± 150.38	57	20	450	117.2
*Fruits and vegetables*						
Pumpkin leaves	137	88.5 ± 89.36	57	4	691	101
Rape relish	84	45.9 ± 49.23	30	9	356	107.2
Rape	35	82 ± 72.23	48	15	313	88.1
Cabbage relish	28	46.8 ± 40.77	38	10	205	87.2
Cabbage	24	52 ± 31.51	44	13	137	60.6
Okra	24	70.7 ± 53.68	54.5	15	245	75.9
Rape soup	13	64 ± 46.69	59	13	184	73
Vegetable relish	10	38 ± 13.15	41.5	17	56	34.6
Banana	10	65.9 ± 51.06	54.5	15	155	77.5
*Meat and fish products*						
Kapenta	21	55.4 ± 59.13	33	9	250	106.7
Chicken	16	42.6 ± 31.79	40.5	9	135	74.7
*Eggs and egg products*						
Egg	12	41.3 ± 23.46	39	11	84	56.9
*Drinks*						
Sweet beer	71	168 ± 79.28	154	43	501	47.2
Soft drinks	57	139.5 ± 83.57	120	10	402	59.9
Tea	15	196.2 ± 104.58	150	84	445	53.3
*Milk and milk products*						
Breast milk	18	38.8 ± 26.21	30	10	96	67.6
Sour milk	12	138.3 ± 70.4	126.5	73	320	50.9

The cutoff point of the frequency is 10 (accounts for 95% of food commonly consumed).

**Table 4:** Food frequency and weight (grams) consumed by children in Monze.

Food type	*N*	Mean ± SD	Median	Min.	Max.	CV
*Cereals and cereal-based products*						
Nshima	159	121.9 ± 65.20	112	25	369	53.5
Maize porridge	145	154.3 ± 82.40	135	32	619	53.4
Cheele	57	216.4 ± 116.36	204	31	565	53.8
Samp	29	108.7 ± 53.09	102	20	214	48.9
*Legumes and legume-based products*						
Beans	38	81.9 ± 38.32	66.5	34	213	46.8
*Fruits and vegetables*						
Rape	51	48.2 ± 28.18	40	18	175	58.5
Okra	36	55.2 ± 19.79	55	22	105	35.8
Cabbage relish	28	46.8 ± 40.77	38	10	205	87.2
Pumpkin leaves	24	69.8 ± 35.56	60	20	164	50.9
Rape relish	23	48.8 ± 56.32	27	12	216	115.3
*Amaranthus*	19	61.0 ± 40.73	59	15	186	66.8
Cabbage	16	48.6 ± 18.33	39.5	24	85	37.7
Black jack	11	62.1 ± 24.76	63	31	104	39.9
*Fish and fish products*						
Fish	12	49.8 ± 57.25	23	10	210	115.1
Kapenta	13	46.1 ± 44.42	36	10	170	96.4
*Eggs and egg products*						
Eggs	13	66.3 ± 58.62	59	11	210	88.4
*Drinks*						
Sweet beer	90	201.3 ± 99.81	190	43	501	49.6
Chibwantu	25	235.4 ± 88.42	236	129	516	37.6
*Milk and milk products*						
Sour milk	12	138.3 ± 70.40	126.5	73	320	50.9

The cutoff point of the frequency is 10 (accounts for 95% of food commonly consumed).

**Table 5:** Food frequency and weight (grams) consumed by mothers in Chipata.

Food type	*N*	Mean ± SD	Median	Min.	Max.	CV
*Cereals and cereal-based products*						
Nshima	218	602.1 ± 248.09	604.5	59	1890	41.2
Porridge(maize)	142	371.9 ± 182.78	360	99	1420	49.1
Buns	80	92.6 ± 81.18	76	18	600	87.6
Fresh maize	33	166.7 ± 161.29	123	27	909	96.8
Rice	17	308.1 ± 169.46	285	132	735	55
*Legumes and legume products*						
Beans	70	183.4 ± 74.02	167.5	58	412	40.4
Soya pieces	39	136.9 ± 137.19	94	22	851	100.2
Cowpea	10	200.3 ± 127.05	143.5	108	500	63.4
Groundnut	31	122.1 ± 84.3	100	16	357	69
*Starchy roots and tubers*						
Sweet potato	29	325.8 ± 195.58	295	104	900	60
Cassava	22	229.7 ± 196.73	173	10	785	85.6
Potato	12	323.3 ± 139.93	285.5	149	613	43.3
*Fruits and vegetables*						
Pumpkin leaves	172	198 ± 109.84	189.5	18	901	55.5
Rape	144	151.2 ± 65.72	136.5	20	341	43.5
Samp	102	495.2 ± 224.45	486	74	1132	45.3
Cabbage	49	172 ± 73.70	168	28	317	42.8
Okra	41	152.9 ± 91.14	137	39	438	59.6
Rape relish	37	132.5 ± 98.43	115	37	630	74.3
Cabbage relish	29	145 ± 74.22	125	47	348	51.2
Vegetable relish	19	170.4 ± 84.36	170	17	321	49.5
Delele	16	167.9 ± 152.2	119.5	47	698	90.7
Banana	13	188.8 ± 176.84	132	10	600	93.7
Mango	11	125 ± 82.53	97	30	290	66
*Meat and fish products*						
Kapenta	40	101.2 ± 76.08	80	18	314	75.2
Chicken	27	82 ± 69.55	52	18	256	84.8
Pork meat	12	53.3 ± 22.68	45.5	26	97	42.5
Fish relish	10	126.1 ± 110.56	82.5	35	346	87.7
*Drinks*						
Soft drink	80	236.3 ± 95.35	244.5	13	385	40.4
Sweet beer	61	519 ± 248.75	444	67	1552	47.9
Chibwantu	10	980.2 ± 333	995.5	546	1564	34
Tea	43	317 ± 130.57	318	25	523	41.2

The cutoff point of the frequency is 10 (accounts for 95% of food commonly consumed).

**Table 6:** Food frequency and weight (grams) consumed by mothers in Monze.

Food type	*N*	Mean ± SD	Median	Min	Max	CV
*Cereals and cereal-based products*						
Nshima	177	650.2 ± 288.28	686	10	1890	44.3
Porridge	24	505.9 ± 213.33	541.5	141	915	42.2
Rice	13	416.9 ± 198.26	356	129	890	47.6
Magwaza	10	314.9 ± 217.79	234	153	833	69.2
Cheele	37	493.2 ± 232.12	468	109	1036	47.1
*Legumes and legume products*						
Beans	48	208.2 ± 76.00	189	62	412	36.5
*Fruits and vegetables*						
Samp	119	515.5 ± 235.54	499	80	1300	45.7
Rape	68	128.1 ± 56.03	110.5	50	310	43.7
Okra	59	145.1 ± 69.53	134	39	438	47.9
Rape relish	45	128.7 ± 94.63	110	37	630	73.5
Cabbage relish	41	146.1 ± 76.25	125	47	348	52.2
Pumpkin leaves	34	183.9 ± 145.12	150	70	901	78.9
Cabbage	26	163.9 ± 79.76	132.5	37	306	48.7
*Amaranthus*	24	163.9 ± 80.87	142	67	383	49.3
Vegetable relish	19	170.4 ± 84.36	170	17	321	49.5
Black jack	11	153.4 ± 47.01	169	80	227	30.7
Okra relish	11	219.4 ± 105.81	207	84	418	48.2
Chiyuniyuni	11	141.5 ± 37.78	137	86	213	26.7
*Drinks*						
Sweet beer	82	568.5 ± 330.21	469	67	1815	58.1
Chibwantu drink	14	671.6 ± 405.68	513.5	363	1600	60.4
*Meat and fish products*						
Kapenta	21	62.8 ± 40.38	50	29	210	64.3
Fish	18	152.8 ± 145.76	104	25	510	95.4
Chicken	13	133.6 ± 65.37	117	54	256	48.9
Fish relish	11	116.5 ± 109.57	55	21	346	94

The cutoff point of the frequency is 10 (accounts for 95% of food commonly consumed).

**Table 7:** Dietary diversity scores (DDSs) for children and mothers.

DDS	Chipata (217)	Monze (177)	Total (394)	*χ* ^2^
*Children*				
Mean	3.9	4.4	4.1	
SD	1.37	1.34	1.38	0.0001
Min.	1	1		
Max.	8	8		
*Scores*				
1	3 (1.38%)	3 (1.69%)	6 (1.52%)	0.004
2	33 (15.21%)	10 (5.65%)	43 (10.91%)	
3	57 (26.27%)	31 (17.51%)	88 (22.34%)	
4	59 (27.19%)	51 (28.81%)	110 (27.92%)	
5	39 (17.97%)	46 (25.99%)	85 (21.57%)	
6	17 (7.83%)	28 (15.83%)	45 (11.42%)	
7	8 (3.69%)	6 (3.39%)	14 (3.55%)	
8	1 (0.46%)	2 (1.13%)	3 (0.76%)	
*Category*				
Low (1–4)	152 (70.05%)	95 (53.67%)	247 (62.69%)	0.0008
High (5–8)	65 (29.95%)	82 (46.33%)	147 (37.31%)	
*Mothers*				
Mean	5.1	4.6	4.8	0.0002
SD	1.47	1.08	1.33	
Min.	2	2		
Max.	8	8		
*Scores*				
2	8 (3.65%)	2 (1.13%)	10 (2.53%)	0.001
3	26 (11.87%)	24 (13.56%)	50 (12.63%)	
4	44 (20.09%)	64 (36.16%)	108 (27.27%)	
5	59 (26.94%)	55 (31.07%)	114 (28.79%)	
6	43 (19.63%)	23 (12.99%)	66 (16.67%)	
7	28 (12.79%)	9 (5.08%)	37 (9.34%)	
8	11 (5.02%)	0 (0.00%)	11 (2.78%)	
*Category*				
Low (1–4)	78 (35.62%)	90 (50.85%)	168 (42.42%)	0.0023
High (5–8)	141 (64.38%)	87 (49.15%)	228 (57.58%)	

**Table 8:** Correlations of DDS of mothers and sociodemographic characteristics of household heads and mothers in Chipata and Monze districts.

DDS	Age of HH	Sex of HH	Marital status of HH	Education of HH	Age of mother	Marital status of mothers	Mothers' education
Chipata (313)	−0.09303^ns^	0.07468^ns^	0.00250^ns^	0.15268^*∗∗*^	−0.16728^*∗∗*^	−0.06189^ns^	0.21265^*∗∗∗*^
Monze (268)	0.15209^*∗*^	—	−0.11688^ns^	0.15271^*∗*^	−0.08110^ns^	−0.03077^ns^	0.04739^ns^

^*∗*^Significant at *p* < 0.05 level. ^*∗∗*^Significant at *p* < 0.01. ^*∗∗∗*^Significant at *p* < 0.001 level. ns, not significant; HH, household head.

## Data Availability

The data related to this manuscript have been deposited in the open access IITA CKAN research data repository.
